# Effects of Acute Resistance Exercise on Executive Function: A Systematic Review of the Moderating Role of Intensity and Executive Function Domain

**DOI:** 10.1186/s40798-022-00527-7

**Published:** 2022-12-08

**Authors:** Tzu-Yu Huang, Feng-Tzu Chen, Ruei-Hong Li, Charles H. Hillman, Trevor L. Cline, Chien-Heng Chu, Tsung-Min Hung, Yu-Kai Chang

**Affiliations:** 1 Department of Physical Education and Sport Sciences, National Taiwan Normal University, 162, Section 1, Heping E. Road., Taipei, 106 Taiwan; 2 Department of Sports Medicine, China Medical University, Taichung, Taiwan; 3 Department of Psychology, Northeastern University, Boston, MA USA; 4 Department of Physical Therapy, Movement, and Rehabilitation Sciences, Northeastern University, Boston, MA USA; 5 Institute for Research Excellence in Learning Science, National Taiwan Normal University, Taipei, Taiwan

**Keywords:** Cognitive function, Inhibition, Resistance exercise, Shifting, Updating

## Abstract

**Background:**

Research has demonstrated that there is a beneficial effect of acute exercise on cognitive function; however, the moderators of the acute resistance exercise (RE) effect on executive function (EF) are underestimated. This systematic review aims to clarify the effects of acute RE on EF by examining the moderating effect of exercise intensity (light, moderate, and vigorous) and EF domains (inhibitory control, working memory, and cognitive flexibility), as well as their interactions.

**Methods:**

The search strategy was conducted in four databases (PubMed, Scopus, PsycARTICLES, and Cochrane Library) prior to January 29, 2022. Included studies had to: (1) investigate acute RE in adults with normal cognition and without diagnosed disease; (2) include a control group or control session for comparison; (3) include outcomes related to the core EF domains; and (4) be published in English. The methodological quality of the included studies was judged according to the PEDro scale guidelines.

**Results:**

Nineteen studies were included which included a total of 692 participants. More than half of the outcomes (24/42, 57.14%) indicate that acute RE had a statistically significant positive effect on overall EF. In terms of RE intensity and EF domain, moderate intensity acute RE benefited EF more consistently than light and vigorous intensity acute RE. Acute RE-induced EF benefits were more often found for inhibitory control than for working memory and cognitive flexibility. When considering moderators simultaneously, measuring inhibitory control after light or moderate intensity RE and measuring working memory or cognitive flexibility after moderate intensity RE most often resulted in statistically significant positive outcomes.

**Conclusion:**

Acute RE has a beneficial effect on EF, observed most consistently for inhibitory control following moderate intensity RE. Future studies should include all exercise intensities and EF domains as well as investigate other potential moderators to enable a better understanding of the benefits of acute RE on EF.

**Supplementary Information:**

The online version contains supplementary material available at 10.1186/s40798-022-00527-7.

## Key Points

- Acute resistance exercise has a beneficial effect on executive function, but the effect is moderated by exercise intensity and executive function domain.
- Light to moderate intensity resistance exercise has a beneficial effect on inhibitory control.
- Moderate intensity resistance exercise has a beneficial effect on working memory and cognitive flexibility.

## Introduction

A growing body of research has demonstrated a beneficial effect of acute exercise on cognition, particularly for tasks or task components with larger executive function (EF) demands [1–5]. Broadly defined, EF refers to a family of cognitive processes that enable the volitional control of thoughts, emotions, attention, and behaviors to complete task-oriented goals [6, 7]. EF includes three core domains: inhibitory control (the ability to make appropriate decisions without being affected by internal tendencies or external distractions), working memory (the ability to store or update specific information in response to task demands), and cognitive flexibility (the ability to use inhibitory control and working memory to alter or redress one’s perspective of and approach to a given situation) [8]. These core EF domains are associated with academic performance, vocational achievement, and positive social relationships [6, 7]. They are also a key determinant of successful aging [9] and efficiency of daily living, an indispensable part of everyday life [10, 11] Accordingly, sustaining and improving EF has become an important public health issue. Critically, previous studies have shown that EF is enhanced following a single bout (i.e., dose) of exercise (also called acute exercise) [1–4].

The recommendations for exercise prescription by the American College of Sports Medicine (ACSM) are centered on two core components: aerobic exercise (AE) and resistance exercise (RE) [12]. While AE improves cardiorespiratory fitness (CRF) through continuous and rhythmic movements, RE increases muscular fitness by utilizing the muscles or muscle groups against external force [12]. Importantly, the differences between AE and RE are responsible for eliciting different physiological responses [13–15]. While a rapidly growing literature has long since established that both acute and chronic AE can benefit EF across the lifespan [16], research into the cognitive (and brain) effects of acute RE has lagged.

Early investigations by Chang and Etnier [17] and Pontifex et al. [18] found that acute RE had beneficial effects on EF. Over the last decade, this area of study has continued to capture our attention. Recent reviews [4, 19] have supported the acute RE-EF link, providing an additional focus on the importance of moderating variables; particularly, exercise intensity and EF domain. However, these recent reviews differ in two key respects. Specifically, Hsieh et al. [19] found that moderate intensity exercise had the most prominent effects on EF, while Wilke et al. [4] found that light and vigorous intensity exercise had positive effects on EF that were not observed at a moderate intensity. Further, Hsieh et al. [19] found benefits of RE across all core EF domains, while Wilke et al. [4] found benefits only for inhibitory control and cognitive flexibility (i.e., not for working memory). Accordingly, the inconsistency between these two reviews requires further examination based on the individual and combined moderating effects of exercise intensity and EF domain. Additionally, there have been numerous novel findings since the review of Wilke et al. [4], warranting further summary and updating of the extant literature to better understand the relationship between acute RE and EF [20–29].

Accordingly, we systematically reviewed the literature on the effects of acute RE on EF. Specifically, we examined the individual and combined moderating effects of exercise intensity (i.e., light, moderate, vigorous) and EF domain (i.e., inhibitory control, working memory, cognitive flexibility) on the acute RE–EF relationship using an analysis to calculate the percentage of positive, negative, and null effects. This systematic review will aid the provision of precise exercise prescriptions to improve specific EF domains and contribute valuable information for theoretical and practical applications.

## Methods

This systematic review was conducted according to the Preferred Reporting Items for Systematic Reviews and Meta-Analyses (PRISMA) guideline [30].

### Search Strategy

Our search strategy included four electronic databases (i.e., PubMed, Scopus, PsycARTICLES, Cochrane Library), and the final search was conducted on January 29, 2022. The search terms were: (acute OR bout OR session OR immediate OR single) AND (exercise OR training OR physical activity) AND (strength OR resistance OR weight) AND (cognitive OR cognition OR executive OR inhibition OR inhibitory control OR self-control OR self-regulation OR fluid intelligence OR interference control OR selective attention OR working memory OR updating OR mental flexibility OR shifting OR switching). Additional relevant articles were also identified from forwarding citation results in Google Scholar and the reference lists of included publications.

### Eligibility Criteria

This systematic review used the Population, Intervention, Comparisons, Outcome, and Study Design (PICOS) principles [31] to explore the effect of acute RE on EF. Therefore, the inclusion criteria for articles in this review were as follows: (Population) the participants targeted were adults aged 18 years or older without any cognitive impairments or physical/mental illness; (Intervention) the studies included a single bout of RE as treatment; (Comparison) the studies included non-exercise or active control condition/group; (Outcome) the studies included at least one EF outcome; and (Study Design) the studies incorporated crossover or parallel-group comparison trials. Additionally, studies were excluded if acute exercise was combined with any other intervention (e.g., nutritional supplementation); if the exercise characteristics (i.e., load, sets, and/or repetitions) were not described; or if EF outcomes were assessed during dual-task performance that would obscure the main effects of RE on EF. This review included all eligible studies published in peer-review articles in English.

### Identification of Eligible Studies

For tracking eligibility status, a Microsoft Excel spreadsheet was employed. Two authors initially identified titles and abstracts for eligible studies according to the PICOS principles. Following the initial process, the authors then screened the full-text article to further check eligibility for inclusion. When consensus among the two reviewers could not be reached, a third individual was included in the process to arbitrate and reach a decision.

### Data Extraction

Of the included studies, authors’ names, publication year, participant’s characteristics (i.e., sample size, age range), study design, prescription of acute RE (i.e., intensity, number of exercises, number of sets, number of repetitions, movement, rest period, speed, duration), the control group/session, the EF domains, and time of EF assessment were extracted. In particular, the intensity was coded as light intensity [30–49% 1-repetition maximum (1-RM)], moderate intensity (50–69% 1-RM) and vigorous intensity (70–84% 1-RM) [12]. The EF assessments were categorized along with the three EF domains: inhibitory control, working memory, and cognitive flexibility (Additional file 1: Table S1). Following the review conducted by McMorris and Hale [32], the majority of the observed effects of acute exercise on cognitive outcomes in adult samples were accounted for by reaction time, rather than accuracy. As such, given that EF effects, characterized by reaction time, are more sensitive than accuracy in whole population [33], we chose to only include this outcome to provide a concise description of this emerging field of study [34, 35]. Further, in instances when the cognitive tests were administered at multiple time points following the acute exercise bout, only the results from the first time point were included. All data were extracted independently by two authors, and any inconsistency was resolved by discussion.

### Quality Assessment

The quality assessment for included articles was conducted using the Physiotherapy Evidence Database (PEDro) scale [36]. Two authors independently judged the bias risks (i.e., low risk, high risk, unclear) for each included article, and discussed their decision with a third author to establish consensus in instances when inconsistency occurred.

It is extremely difficult to double blind exercise interventions such that both participants and investigators are naïve to the group assignment. As such, this systematic review removed the blinding of participants and investigators similar to a previous meta-analysis [2]. In summary, the final scale of quality assessment in the review was as follows: (1) eligibility criteria; (2) random allocation/counterbalanced order; (3) allocation concealed; (4) similar at baseline; (5) measures of the outcome obtained from > 85% of subjects; (6) intent to treat; (7) between/condition statistical comparisons; and (8) point measure and measures of variability. All included studies were judged for risk of bias levels (i.e., low risk, high risk, unclear risk). The quality rating was performed to present (1) the percentage of these eight quality criteria in different risk of bias levels; and (2) the risk of bias related to individual studies.

## Results

### Selected Studies

**Fig. 1:**
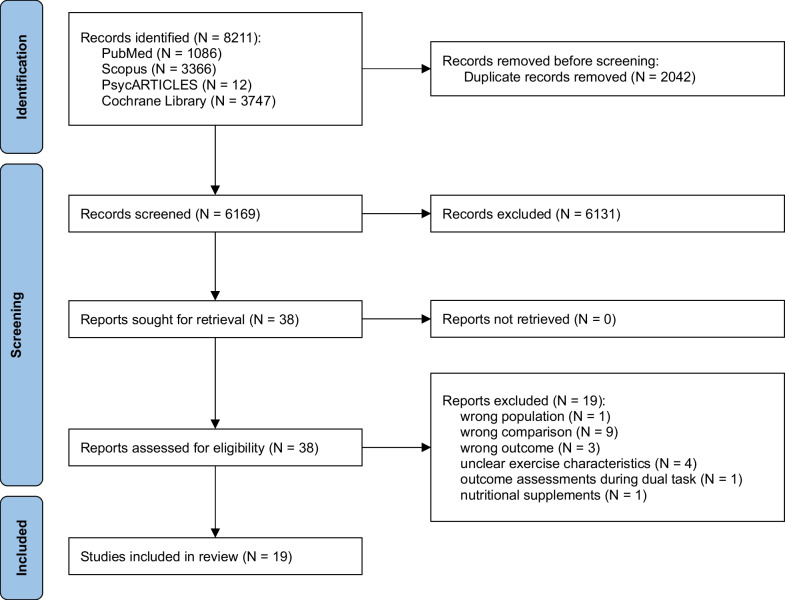
PRISMA study flow diagram of study selection

Figure 1 depicts the process of identifying eligible studies via a PRISMA flowchart. Initially, 8211 articles were identified from the electronic database. After removing 2042 duplicate articles, 6169 articles remained for the title and abstract screening. Then, 6131 articles were removed because they did not meet the study criteria, and the remaining 38 articles were selected for full-text review. Of these studies, we removed 13 articles for not meeting inclusion criteria [37–49], 4 articles for not providing clear exercise characteristics [50–53], 1 article for nutritional supplements intervention [54], and 1 article for assessing EF during dual-task performance [55]. Finally, a total of 19 articles were included in this systematic review (see Fig. 1).

### Study Characteristics

The study characteristics of included articles are summarized in Table 1. The included studies were published between 2009 and 2022. Across all 19 articles, we report data from a total of 692 participants (20–68 years old).

**Table 1:** Overview of studies included for the investigation of the effects of acute resistance exercise on executive function

Author (year)	Subjects *n* (males) Age mean ± SD	Design	Prescription of resistance exercise				Control	EF domain	Timing of exam. (after RE)
			Intensities Exercises × sets × repetitions			Movement Rest periods (Ex., Set.) Speed (Con: Ecc) Duration			
			Low	Moderate	High				
Alves et al. [56]	*n* = 42 (0) 52 ± 7 yr	Crossover		65% 1-RM 6 × 2 × 15		Upper + lower 60 s, 60 s NR 30 min	Reading + stretching	IC	NR
Brush et al. [20]	*n* = 28 (14) 21 ± 1 yr	Crossover	30% 1-RM 7 × 3 × 10	52.5% 1-RM 7 × 3 × 10	75% 1-RM 7 × 3 × 10	Upper + lower 120 s, 120 s NR 45 min	Watching video	IC WM CF	15 min 180 min
Chang and Etnier [17]	*n* = 41 (14) 49 ± 9 yr	Parallel-group			75% 1-RM 6 × 2 × 10	Upper NR NR 45 min	Reading	IC CF	immediate
Chang et al. [57]	*n* = 30 (15) 58 ± 3 yr	Crossover		52.5% 1-RM 7 × 2 × 10		Upper + lower 30 s, 60 s NR 20–25 min	Reading	IC	NR
Chang et al. [62]	*n* = 36 (0) 21 ± 2 yr	Parallel-group			80% 1 RM 7 × 3 × 8–10	Upper + lower 1: 2 NR NR	Seated rest	IC	15 min
Chou et al. [21]	*n* = 70 (31) 47 ± 6 yr	parallel-group			70% 1-RM 7 × 2 × 10	upper + lower 60 s, 60 s NR 20–25 min	reading	IC	Immediate 40 min
de Almeida et al. [29]	*n* = 15 (3) 68 ± 4 yr	Crossover		50% 1-RM 1 × 10 × 12	70% 1-RM 1 × 10 × 12	Lower 90 s* NR 25 min	Reading	IC CF	Immediate
Dunsky et al. [58]	*n* = 39 (29) 52 ± 8 yr	Crossover			75% 1-RM 6 × 3 × 10	Upper + lower 60 s, 60 s con. + ecc. = 2 s 25 min	Watching video	IC	3 min
Hsieh et al. [27]	*n* = 18 (18) 24 ± 2 yr	Crossover		52.5% 1-RM 8 × 2 × 10		Upper + lower 30 s, 90 s NR 30 min	Reading	IC	10 min
	*n* = 17 (17) 66 ± 1 yr								
Hsieh et al. [59]	*n* = 20 (20) 24 ± 2 yr	Crossover		52.5% 1-RM 8 × 2 × 10		Upper + lower 30 s, 90 s NR 30 min	Reading	WM	10 min
	*n* = 20 (20) 67 ± 2 yr								
Lin et al. [22]	*n* = 28 (28) 60 ± 4 yr	Crossover			75% 1-RM 3 × 3 × 5	Upper + lower 120–180 s, NR 2 s: 2 s 30 min	Stretching	IC	10 min
Naderi et al. [23]	*n* = 48 (24) 64 ± 3 yr	Parallel-group (exercise) + crossover (intensities)	30% 1-RM 8 × 3 × 10	52.5% 1-RM 8 × 3 × 10		upper + lower 30 s, 90 s NR 45 min	Watching video	IC WM CF	15 min 180 min
Palmiere et al. [28]	*n* = 35 (16) 22 ± 3 yr	Crossover			75% 1-RM 1 × 5 × 10 + 87% 1-RM 1 × 5 × 5	Upper 90 s, NR NR 30 min	Watching video	IC WM	10–30 min
Pontifex et al. [18]	*n* = 21 (12) 20 ± 1 yr	Crossover			80% 1-RM 7 × 3 × 8–12	Upper + lower 60 s, 90 s NR 30 min	Seated rest	WM	Immediate 30 min
Tsai et al. [61]	*n* = 60 (60) 23 ± 2 yr	Parallel-group		50% 1-RM 6 × 2 × 10	80% 1-RM 6 × 2 × 10	Upper + lower 90 s, 120 s NR 30 min	Reading	IC	5 min
Tsuk et al. [24]	*n* = 40 (19) 26 ± 3 yr	Crossover		60% 1-RM 6 × 3 × 15		Upper + Lower 60 s, NR NR 30 min	Seated rest	IC	3 min
Tsukamoto et al. [60]	*n* = 12 (12) 23 ± 1 yr	Crossover	40% 1-RM 1 × 6 × 10		80% 1-RM 1 × 6 × 10	Lower 180 s* 1 s: 1 s 17 min	Seated rest	IC	Immediate
Wang et al. [25]	*n* = 42 (25) 21 ± 1 yr	Crossover			70% 1-RM 7 × 2 × 8–12	Upper + lower 30 s, 60 s NR 20 min	Reading	IC	10 min
Wu et al. [26]	*n* = 30 (17) 21 ± 1 yr	Crossover		52.5% 1-RM 7 × 2 × 8–12		Upper + lower NR NR 20 min	Reading	CF	30 min

*yr*, years; *Ex*., rest periods between exercises; *Set*., rest periods between sets; *, one exercise movement only (i.e., without rest periods between exercises); *Con*, concentric contraction; *Ecc*, eccentric contraction; *exam*, examination; *RM*, repetition maximum; *RE*, resistance exercise; *Upper*, upper limbs; *Lower*, lower limbs; *NR*, not reported; *EF*, executive function; *IC*, Inhibitory control; *WM*, working memory; *CF*, cognitive flexibility

Study design: 14 studies included a within-subject design [18, 20, 22, 24–29, 56–60] and 5 studies included a between-subject design [17, 21, 23, 61, 62].

Method of movement in acute RE: 2 studies included only upper body [17, 28], 2 studies included only lower body [29, 60], and 15 studies included combining upper and lower body [18, 20–27, 56–59, 61, 62]. Additionally, the duration of acute RE was between 17 and 45 min.

Intensity of acute RE: 3 studies used light-intensity treatment [20, 23, 60], 10 studies used moderate-intensity treatment [20, 23, 24, 26, 27, 29, 56, 57, 59, 61], and 12 studies used vigorous-intensity treatment [17, 18, 20–22, 25, 28, 29, 58, 60–62]. Of these, 4 studies compared two categories of intensity [20, 23, 60, 61] and 1 study compared all three categories of intensity [20].

EF domains: 13 studies focused on one domain of EF [18, 21, 22, 24–27, 57–62] and 6 studies focused on two or more domains of EF [17, 20, 23, 28, 29, 56]. Of these, 16 studies examined inhibitory control [17, 20–25, 27–29, 56–58, 60–62], 5 studies examined working memory [18, 20, 23, 28, 59], and 6 studies examined cognitive flexibility [17, 20, 23, 26, 29, 56].

### Quality Assessment

**Fig. 2:**
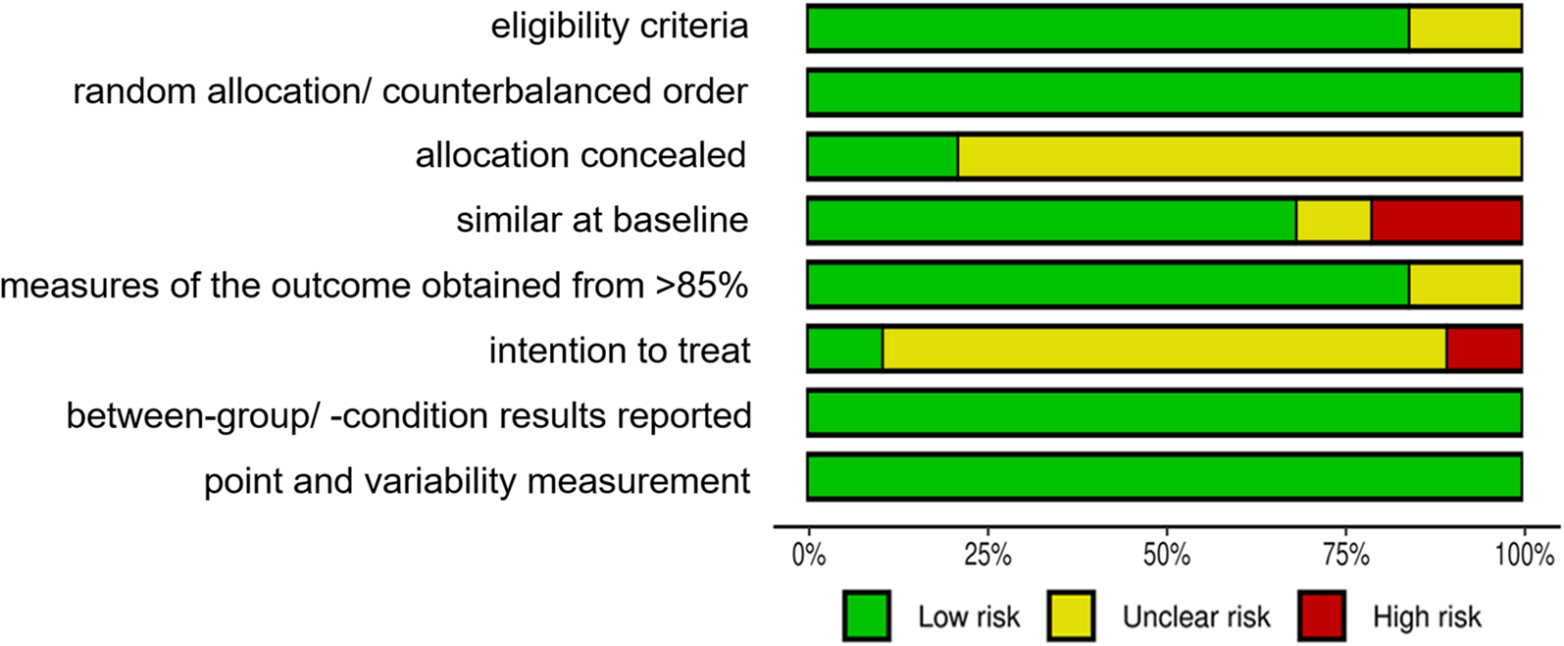
Overview of the revised PEDro rated study quality

The initial level of agreement between the two raters was high (Cohen’s kappa = 0.98). All discrepancies were resolved by discussion without consulting a third investigator. On average, the studies reached good methodological quality. All studies were judged low risk for random allocation, between/condition comparison, and both point estimates measures. Most studies were judged low risk for eligibility criteria, baseline comparability and proper continuation. Only 4 studies (21.05%) were judged low risk for concealed allocation [17, 20, 21, 58], and 2 studies (10.52%) were judged low risk for intent to treat [22, 58]. The overview of quality assessment is presented in Fig. 2. More detailed information on individual ratings can be found in Additional file 1: Fig. S1.

### Main Results

Tables 2 and 3 provide a summary of the overall results and moderating effects of the 19 included studies. A total of 42 outcomes were extracted for comparison between the acute RE group/session and control group/session. Overall, 24 (57.14%) outcomes indicated positive effects, 18 outcomes (42.86%) indicated null effects, and no outcomes (0.00%) indicated negative effects for acute RE-induced changes in EF task performance.

**Table 2:** Results of the overall effect of acute resistance exercise on executive function and the moderating effects of exercise intensity and EF domain

	*n* of outcomes	*n* of positive effects	Positive effect (%)
Overall	42	24	57.14
*Exercise intensity*			
Light	7	4	57.14
Moderate	18	13	72.22
Vigorous	17	7	41.17
*EF sub-domain*			
Inhibitory control (IC)	23	15	65.21
Working memory (WM)	9	4	44.44
Cognitive flexibility (CF)	10	5	50.00
*Combined moderating effects*			
IC, light	3	2	66.67
IC, moderate	9	7	77.78
IC, vigorous	11	6	54.54
WM, light	2	1	50.00
WM, moderate	4	3	75.00
WM, vigorous	3	0	0.00
CF, light	2	1	50.00
CF, moderate	5	3	60.00
CF, vigorous	3	1	33.33

**Table 3:** Individual outcomes for the moderating effects of exercise intensity and EF domain

Intensity	Authors	% 1-RM	IC	WM	CF
**Light (30–49% 1-RM)**	Brush et al. [20]	30	0	0	0
	Naderi et al. [23]	30	1	1	1
	Tsukamoto et al. [60]	40	1	–	–
	Number of positive effects		2	1	1
	Number of outcomes		3	2	2
	Positive effect (%)		66.67	50.00	50.00
**Moderate (50–69% 1-RM)**	de Almeida et al. [29]	50	1	–	1
	Tsai et al. [61]	50	0	–	–
	Brush et al. [20]	52.5	0	0	0
	Chang et al. [57]	52.5	1	–	–
	Hsieh et al. -1 [27]*	52.5	1	–	–
	Hsieh et al. -2 [27]*	52.5	1	–	–
	Hsieh et al. -1 [59]*	52.5	–	1	–
	Hsieh et al. -2 [59]*	52.5	–	1	–
	Naderi et al. [23]	52.5	1	1	1
	Wu et al. [26]	52.5	–	–	1
	Tsuk et al. [24]	60	1	–	–
	Alves et al. [56]	65	1	–	0
	Number of positive effects		7	3	3
	Number of outcomes		9	4	5
	Positive effect (%)		77.78	75.00	60.00
**Vigorous (70–84% 1-RM)**	Chou et al. [21]	70	1	–	–
	de Almeida et al. [29]	70	1	–	1
	Wang et al. [25]	70	1	–	–
	Brush et al. [20]	75	1	0	0
	Chang and Etnier [17]	75	0	–	0
	Dunsky et al. [58]	75	1	–	–
	Lin et al. [22]	75	0	–	–
	Palmiere et al. [28]	75	0	0	–
	Chang et al. [62]	80	0	–	–
	Pontifex et al. [18]	80	–	0	–
	Tsai et al. [61]	80	0	–	–
	Tsukamoto et al. [60]	80	1	–	–
	Number of positive effects		6	0	1
	Number of outcomes		11	3	3
	Positive effect (%)		54.54	0.00	33.33

1, positive effect; 0, null effect

*The study consists of two trials (i.e., numbers 1 and 2); *IC*, Inhibitory control; *WM*, working memory; *CF*, cognitive flexibility

Of the included studies for the moderating effects of exercise intensity, 4 of 7 outcomes (57.14%) for light intensity; 13 of 18 outcomes (72.22%) for moderate intensity; and 7 of 17 outcomes (41.17%) for vigorous intensity were found to significantly improve EF following an acute bout of RE.

Of the included studies for moderating effects of EF domains, 15 of 23 outcomes (65.21%) for inhibitory control; 4 of 9 outcomes (44.44%) for working memory; and 5 of 10 outcomes (50.00%) for cognitive flexibility were found to be significantly improved following an acute bout of RE.

Of the included studies for the combined moderating effects of exercise intensity and EF domain on inhibitory control, 2 of 3 outcomes (66.67%) for light intensity; 7 of 9 outcomes (77.78%) for moderate intensity; and 6 of 11 outcomes (54.54%) for vigorous intensity were found to significantly improve inhibitory control following an acute bout of RE. Of the included studies reporting on working memory, 1 of 2 outcomes (50.00%) for light intensity; 3 of 4 outcomes (75.00%) for moderate intensity; and no outcomes (0/3, 0.00%) for vigorous intensity were found to significantly improve working memory following an acute bout of RE. Of the included studies reporting on cognitive flexibility, 1 of 2 outcomes (50.00%) for light intensity; 3 of 5 outcomes (60.00%) for moderate intensity; and 1 of 3 outcomes (33.33%) for vigorous intensity were found to significantly improve cognitive flexibility following an acute bout of RE.

## Discussion

This systematic review investigated the effects of acute RE on EF and further examined the moderating effects of exercise intensity, EF domain, and their interaction. Based on 42 outcomes across 19 studies, 57.14%, an overall positive effect of acute RE on EF was observed; however, we should note that 42.86% observed a null effect. The relatively similar percentage of positive (57.14%) and non-significant (42.86%) results further emphasizes the importance of analyzing potential moderating variables, particularly exercise intensity, EF domains, and the combination of these two variables, to better understand the relationship of RE on EF.

### The Moderating Effects of Exercise Intensity

Although some of the included studies did not delineate the effects of specific exercise intensity, our results show that no study found acute RE (at any intensity) had a detrimental effect on EF. Specifically, moderate intensity exercise was most consistently found to confer EF benefits (13/18, 72.22% outcomes had a positive effect). In contrast, light (4/7, 57.14%) and vigorous intensity (7/17, 41.17%) RE were related to EF benefits in approximately half of the included outcomes. These findings differ from the review by Wilke et al. [4], which extracted results of EF 5 min after acute exercise and found that only light and vigorous (but not moderate) intensity acute RE had positive moderating effects. Furthermore, these inconsistent results might stem from our broader criterion for the timing of cognitive testing after acute RE (0–30 min), which led to the inclusion of more studies in our analysis (*N* = 19) relative to Wilke et al. [4], affording us the opportunity to provide a broader review of the extant literature. Therefore, we suggest based on our summary that the timing of cognitive testing is a leading cause of the inconsistent results observed across the two systematic reviews.

The moderating effect of RE intensity on EF outcomes may be related to physiological processes resultant from exercise. Arousal level is frequently identified as a potential mechanism underlying the effects of acute exercise on EF [63–65]. Dose–response studies have illustrated an inverted-U shaped relationship between acute RE and cognitive performance, with optimal effects occurring at moderate RE intensity (i.e., moderate levels of arousal) [47, 66]. Previous studies have suggested that increases in arousal are related to upregulation of endocrine activity, including elevations in plasma concentrations of epinephrine, dopamine, and norepinephrine [67] activity, as well as activation of the locus coeruleus-norepinephrine system (LC-NE system) [68, 69]. Importantly, recent research indicates that the LC-NE system may be regulated, in part, by acute RE, and exhibits an inverted-U relationship with cognitive outcomes [70]. These findings provide mechanistic support for the acute RE relationship with EF via facilitation of endocrine activation regulating arousal level.

Another often-proposed mechanism underlying the acute exercise–EF relationship suggests that neurotrophic factors such as BDNF and IGF-1 may regulate the observed effects, which aid in the promotion and maintenance of neuronal health and synaptic proliferation [5]. Marston et al. [71] reviewed seven studies on acute RE and BDNF and found that acute vigorous RE elevated peripheral BDNF levels. However, only Tsai et al. [72] examined the association among RE, BDNF, and cognitive function, and found no significant changes in peripheral BDNF levels after moderate intensity acute RE in older adults with mild cognitive impairment. Whether a similar relationship (or lack thereof) may emerge in healthy adults remains to be investigated. With regard to IGF-1, Tsai et al. [61] found that peripheral IGF-1 levels were significantly increased following acute RE, but no associations were found with cognitive performance. While it is important to remember that peripheral growth factor measurements are not necessarily reflective of concentrations in the central nervous system, the current evidence has not provided consistent support for growth factor regulation as a potential mechanism underlying acute RE and cognition. Accordingly, the positive cognitive effects observed following acute resistance exercise may be underpinned by several endocrine and molecular mechanisms that are influenced by exercise intensity. However, our understanding of these biological mechanisms is still limited, and more studies binding the biological processes are needed to better determine the underlying mechanisms giving rise to acute RE effects on cognitive outcomes.

### The Moderating Effects of Core EF

The majority of studies in this systematic review investigated inhibitory control outcomes, which were also found to have the largest percentage of statistically significant positive effects among the three EF domains (15/23, 65.21%). Comparatively, 44.44% (4/9) and 50.00% (5/10) of the studies reported positive effects on working memory and cognitive flexibility, respectively. This finding is consistent, in part, with the results of Wilke et al. [4], who found that acute RE positively affected inhibitory control and cognitive flexibility, but not working memory. Interestingly, similar results have been found in a study on acute high-intensity interval training (HIIT), which demonstrated positive effects on inhibitory control [34]. However, the finding should be interpreted with caution as over half of the outcomes reported in these reviews focused on inhibitory control, which might produce a statistical bias when comparing RE effect across all three core EF domains.

Yet, the evidence for inhibitory control is interesting given that it is considered to be the “purest” of the three core EF domains, and important for the application of working memory and cognitive flexibility [73]. As Miyake et al. [74] noted, although inhibitory control, working memory, and cognitive flexibility are correlated with each other, they remain distinctive aspects of EF. If the benefit to EF induced by acute exercise is preferential to inhibitory control, this may be reflected in the neural system underlying this cognitive process. While EF tasks are associated with a broad neural network spanning the cortex (e.g., prefrontal, parietal, anterior cingulate cortices), distinct regions associated with each EF domain are observable in the functional activation patterns [75], and damage to different regions of the frontal lobe differentially affects core EF domains [11]. Interestingly, reduced grey matter volume in the medial prefrontal cortex and reduced white matter integrity of tracts connecting areas of the frontal lobe (e.g., anterior cingulate, dorsolateral prefrontal cortices) have been associated with age-related declines in inhibitory control [76]. However, even inhibitory control should not be thought of as a unitary cognitive process. As latent variable analysis shows that inhibitory control is further dissociable according to its application to internally or externally directed aspects of inhibition [77], and different tasks of inhibitory control have been associated with overlapping but distinct functional architecture [78]. It is therefore important to consider the network perspective [79] in neurocognitive investigations of exercise. For example, acute AE has been shown to modulate resting-state functional connectivity (rsFC) of cortical networks that may underlie attention- and EF-related improvements [80]. This effect may be further moderated by exercise intensity. Changes in both rsFC and cognitive performance have been found to depend on AE intensity [81], and acute HIIT has been found to simultaneously modulate neural activity (i.e., P3-ERP amplitude and latency) and improve performance on EF tasks [82].

Importantly, this systematic review identified no investigations of acute RE-induced neural modulation associated with specific EF outcomes. The present review therefore encourages future research directed toward the examination of all EF domains and their respective neural underpinnings. Regardless, our findings suggest that inhibitory control may be central to the observed acute exercise-induced EF improvements reported in the literature and calls for more neurocognitive investigations to support or refute this potential selectivity.

### The Combined Moderating Effects of Exercise Intensity and EF Domain

In addition to examining the moderating effects of exercise intensity and specific EF domain, we also evaluated both moderation analyses simultaneously. Importantly, we found that acute RE was observed to positively affect inhibitory control at every exercise intensity. We further found trends suggesting that moderate intensity RE was more consistently observed to have a statistically significant positive effect on EF (7/9, 77.78%) than light (2/3, 66.67%) or vigorous (6/11, 54.54%) intensity RE. Thus, the present results indicate that the strongest combined moderating effects of exercise intensity and EF domain may be observed for inhibitory control processes following a bout of moderate intensity RE.

The effects of RE-induced improvements on working memory were not consistently observed across all exercise intensities. Most studies (3/4, 75.00%) found that the effect of RE on working memory was positive for moderate intensity interventions. Such effects were less consistently observed following light intensity exercise (1/2, 50.00%) and not observed at vigorous intensity exercise (0/3, 0.00%). Similar findings are apparent across studies examining the effects of RE on cognitive flexibility, with the combined moderating effects of cognitive flexibility and intensity most frequently reported at moderate intensity (3/5, 60.00%). In contrast, light (1/2, 50.00%) and vigorous (1/3, 33.33%) intensity RE had relatively less consistent effects on cognitive flexibility. These findings highlight the importance of considering the appropriate intensity when targeting specific EF domains in exercise interventions. However, at present, the findings pertaining to light intensity and vigorous intensity interventions on working memory and cognitive flexibility are scarce (*n* ≤ 3), and we are therefore limited in our ability to draw firm conclusions.

### Inconsistencies in The Literature

In our review, we sought to clarify the independent and combined moderating effects of exercise intensity and EF domain on the acute RE–EF relationship. However, we report that our findings revealed inconsistencies across the included studies that informed our interpretation. Due to the concerns presented here, it is possible that factors other than intensity and EF domain, such as experimental design (e.g., task design, study parameters, testing time, etc.), participant characteristics (e.g., age, sex, etc.), and exercise prescription (e.g., rest periods between sets, mode, movements performed, etc.), may also play important roles in the RE–EF relationship. Nonetheless, with regard to the moderators we examined, there are still several interesting findings. Specifically, the classification of RE intensity level in previous research is diverse. The ACSM guidelines define moderate intensity RE as 50–69% of 1-RM [12], while the same level was coded as 50–75% of 1 RM in Wilke et al. (2019) [4], and 71–80% of 1-RM in Oberste et al. [3]. Such differences make the integrations and comparisons between studies challenging. Furthermore, the most widely used measure of intensity in the included studies, RM, also differed in terms of measurement approach and unit. The units of resistance intervention include 1-RM, 5-RM, 10-RM, and 15-RM. Critically, RM may be directly measured or estimated through multiple repetitions, but previous reports suggest that differences in measurement accuracy for 1-RM estimation can lead to increased statistical error, which could impact results [83].

Additionally, RE prescriptions are comprised of many other considerations [84], with different intensities potentially increasing the diversity of RE outcomes. Specifically, load (as measured by the maximum number of repetitions) was often used to measure the intensity of RE in studies included in our review. However, measuring RE intensity with load does not account for numerous exercise parameters, such as the number of repetitions performed, repetition speed, and the length of rest intervals between sets. Thus, it is difficult to determine the effects of specific intensities of RE on EF outcomes as well as potential underlying physiological mechanisms [85]. While the benefits of exercise are mainly determined by variations in physiological and psychological factors, the use of load is not likely to fully reflect variations in physiological and psychological performance [86]. For example, the ratings of perceived exertion (RPE) is an effective tool, not only as a measurement of exercise intensity, but also as a psychophysiological integrator that can be determined by exercise capacity [87]. Based on the included studies, RPE in moderate intensity interventions were between 11.2 ± 1.8 (light) [20] and 14.9 ± 1.3 (hard/heavy) [57]. These differences in RPE may imply different underlying physiological responses to apparently similar acute RE intensities. It is therefore imperative that future studies consider the nuances of exercise prescriptions that may impact the replicability of neurocognitive investigations of acute RE. In recent years, training volume [43], movement speed [38], and training type (e.g., equipment-based or free weights) [45] have been used to better describe specific aspects of the RE–EF relationship. Through careful continued investigation, the field can broaden the knowledge-base and obtain specific exercise prescriptions for targeted interventions.

### Limitations and Future Directions

Because neurocognitive studies of acute RE are currently in their initial stages, with less than 20 studies focusing on this relationship, the following limitations should be noted. First, this systematic review examined only the moderating effects of exercise intensity and core EF domains. As such, it remains unclear whether other latent variables (e.g., the time of testing, study sample differences, RE designs, etc.) may also moderate the benefits of acute RE. Second, following the review conducted by McMorris and Hale [32] that revealed reaction time accounted for most observed effects of acute exercise in adult samples, it should be noted that we only included reaction time as an indicator of EF and did not investigate response accuracy in the assessment. Third, a relatively low number of studies (*n* ≤ 3) were included that reported relationships between exercise intensity, working memory, and cognitive flexibility, which has the potential to reduce the reliability of our results. Finally, similar to the review conducted by Wilke et al. [4], which used exercise load as an indicator of exercise intensity, this review was unable to account for exercise parameters such as volume, repetition frequency, rest period, and duration.

Although this systematic review focused on the moderating effects of exercise intensity and core EF domains, the inconsistencies in our results could not be fully explained by these two moderating variables. Therefore, future reviews should include additional variables in their analyses, such as experimental design, participant characteristics, and other details of the exercise prescription, as well as the interactions among these variables. Further, few studies directly compared the effects of all three intensities or measured all three core EF domains. As such, more research is needed to better understand the exercise-induced benefits for EF and the implications for brain-health exercise prescription. Furthermore, studies on the physiological mechanisms underlying the effects of acute RE on EF are currently only in the preliminary stages. We expect that further research will more carefully investigate exercise prescription parameters on cognition to better understand the underlying mechanisms. That is, RE programs depend on many parameters, all of which affect the degree of the RE training stimulus. Thus, future studies should consider these parameters to better examine the overall effect of RE on cognitive outcomes.

## Conclusions

This systematic review investigated the effects of acute RE on core EF and examined the independent and combined moderating effects of exercise intensity and EF domain. We report that more than half of the studies included in our systematic review supported a positive effect of acute RE on EF. Moderate intensity exercise was observed more often than light and vigorous intensity exercise to show the largest percentage of statistically significant EF benefit compared to the other intensities. Of the three core EF domains, we report that inhibitory control was most often included as an EF outcome and was likewise found to have the largest percentage of positive outcomes from acute RE. However, a considerably smaller literature on the acute RE effects on working memory and cognitive flexibility exists. When examining the combined moderating effects of exercise intensity and EF, we found that inhibitory control following light and moderate intensity acute RE; working memory following moderate intensity acute RE; and cognitive flexibility following moderate intensity acute RE are the most prominent positive relationships reported in the literature.

## Supplementary Information

**Additional file 1: Table S1**. Classification of executive function assessments. **Table S2**. Quality ratings of individual studies

## Data Availability

Not applicable.
