# Supplementary material for: Effects of Acute Resistance Exercise on Executive Function: A Systematic Review of the Moderating Role of Intensity and Executive Function Domain
Source: Sports Med Open. 2022 Dec 8;8:141. doi: 10.1186/s40798-022-00527-7 (PMC9732176; doi:10.1186/s40798-022-00527-7)
Supplement: Supplementary file 1 — Additional file 1: Table S1. Classification of executive function assessments. Table S2. Quality ratings of individual studies [file 40798_2022_527_MOESM1_ESM.docx]

**Supplementary Material**

| **Article Title** | Effects of Acute Resistance Exercise on Executive Function: A Systematic Review of the Moderating Role of Intensity and Executive Function Domain |
| --- | --- |
| **Authors** | Tzu‑Yu Huang, Feng‑Tzu Chen, Ruei‑Hong Li, Charles H. Hillman, Trevor L. Cline, Chien‑Heng Chu, Tsung‑Min Hung and Yu‑Kai Chang |
| **Contents** | Table S1. Classification of executive function assessments  Fig. S1. Quality ratings of individual studies |

Table S1. Classification of executive function assessments

| **Inhibitory control** | **Working memory** | **Cognitive flexibility** |
| --- | --- | --- |
| Stroop task - color-word  Stroop task -incongruent  Stroop task -interference score  Simon task  Eriksen Flanker task - incongruent  Go/no-go Task | N-back (2 back)  Verbal running span task  Paced auditory serial Addition task  Modified Sternberg test | Trail making test – B  Plus-minus task  Dimension-switching task  More-odd task  Task-switching test |


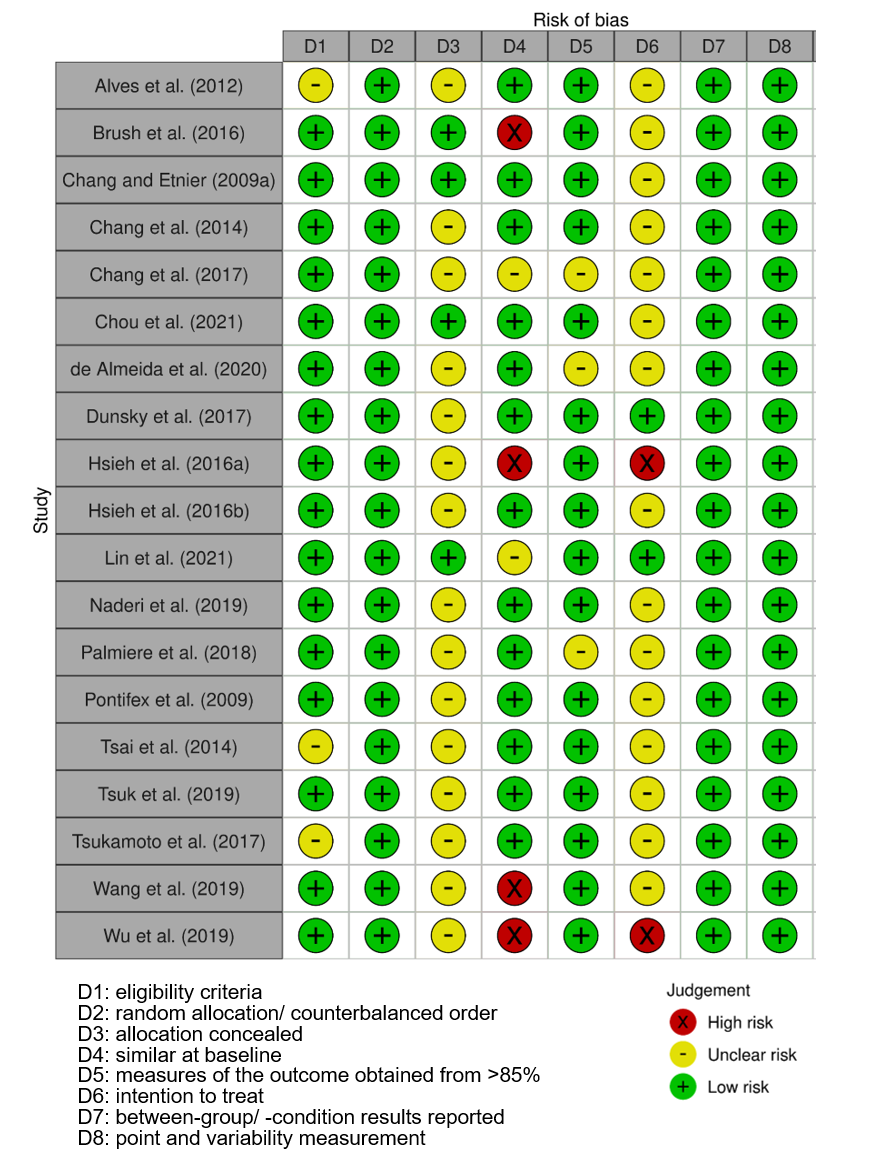


Fig. S1. Quality ratings of individual studies
